# Supplementary material for: Recognition of host Clr-b by the inhibitory NKR-P1B receptor provides a basis for missing-self recognition
Source: Nat Commun. 2018 Nov 5;9:4623. doi: 10.1038/s41467-018-06989-2 (PMC6218473; doi:10.1038/s41467-018-06989-2)
Supplement: Supplementary file 1 — Supplementary Information [file 41467_2018_6989_MOESM1_ESM.pdf]

Recognition of host Clr-b by the inhibitory NKR-P1B receptor provides a basis for missing-self recognition

Gautham R. Balaji[1,2#], Oscar A. Aguilar[3,4#], Miho Tanaka[3], Miguel A. Shingu-Vazquez[1,2], Zhihui Fu[1,2], Benjamin S. Gully[1,2], Lewis L. Lanier[4], James R. Carlyle[3*], Jamie Rossjohn[1,2,5*] & Richard Berry[1,2*]

[1]Infection and Immunity Program and Department of Biochemistry and Molecular Biology, Biomedicine Discovery Institute, Monash University, Clayton, Victoria 3800, Australia
[2]ARC Centre of Excellence in Advanced Molecular Imaging, Monash University, Clayton, Victoria 3800, Australia
[3]Department of Immunology, University of Toronto, and Sunnybrook Research Institute, Toronto, ON, M4N 3M5, Canada
[4]Department of Microbiology and Immunology, University of California, San Francisco, CA, USA and Parker Institute for Cancer Immunotherapy, University of California, San Francisco, CA 94143, USA
[5]Institute of Infection and Immunity, Cardiff University School of Medicine, Heath Park, Cardiff CF14 4XN, U.K.

[#]Co-first authors. [*]Jointly supervising authors. Correspondence and request for materials should be addressed to Richard Berry (Richard.berry@monash.edu), Jamie Rossjohn (Jamie.rossjohn@monash.edu) or James Carlyle (james.carlyle@utoronto.ca)

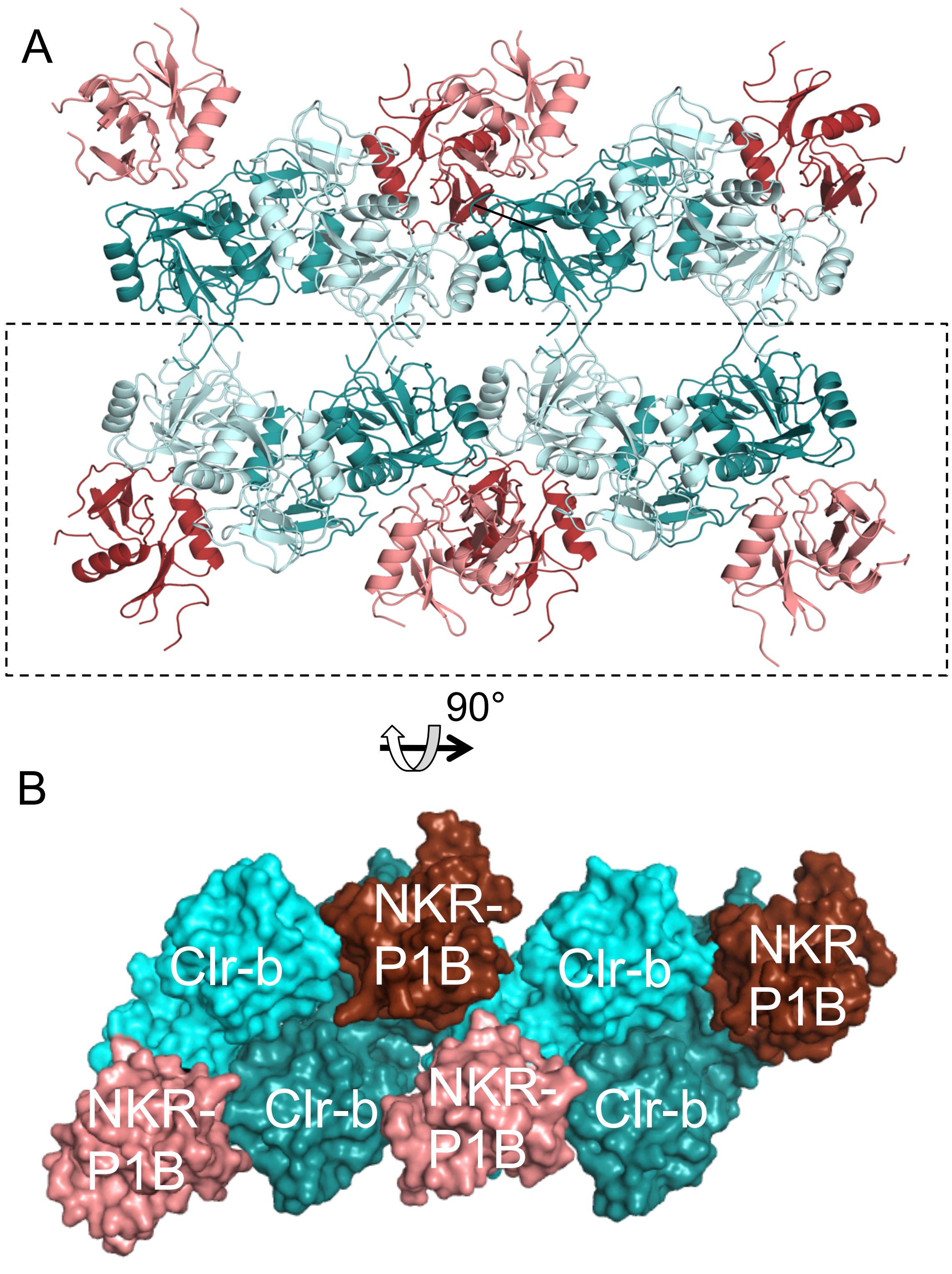

Supplementary Fig. 1. The NKR-P1B:Clr-b asymmetric unit. (A) Overview of the 8 NKR-P1B:Clr-b complexes visible within the crystallographic asymmetric unit. NKR-P1B is shown in salmon, while Clr-b is colored cyan. To aid in visualization, alternating NKR-P1B:Clr-b 'complexes' are colored in light and dark shades respectively. (B) Surface representation showing a top view of half of the crystallographic asymmetric unit (boxed region in (A).

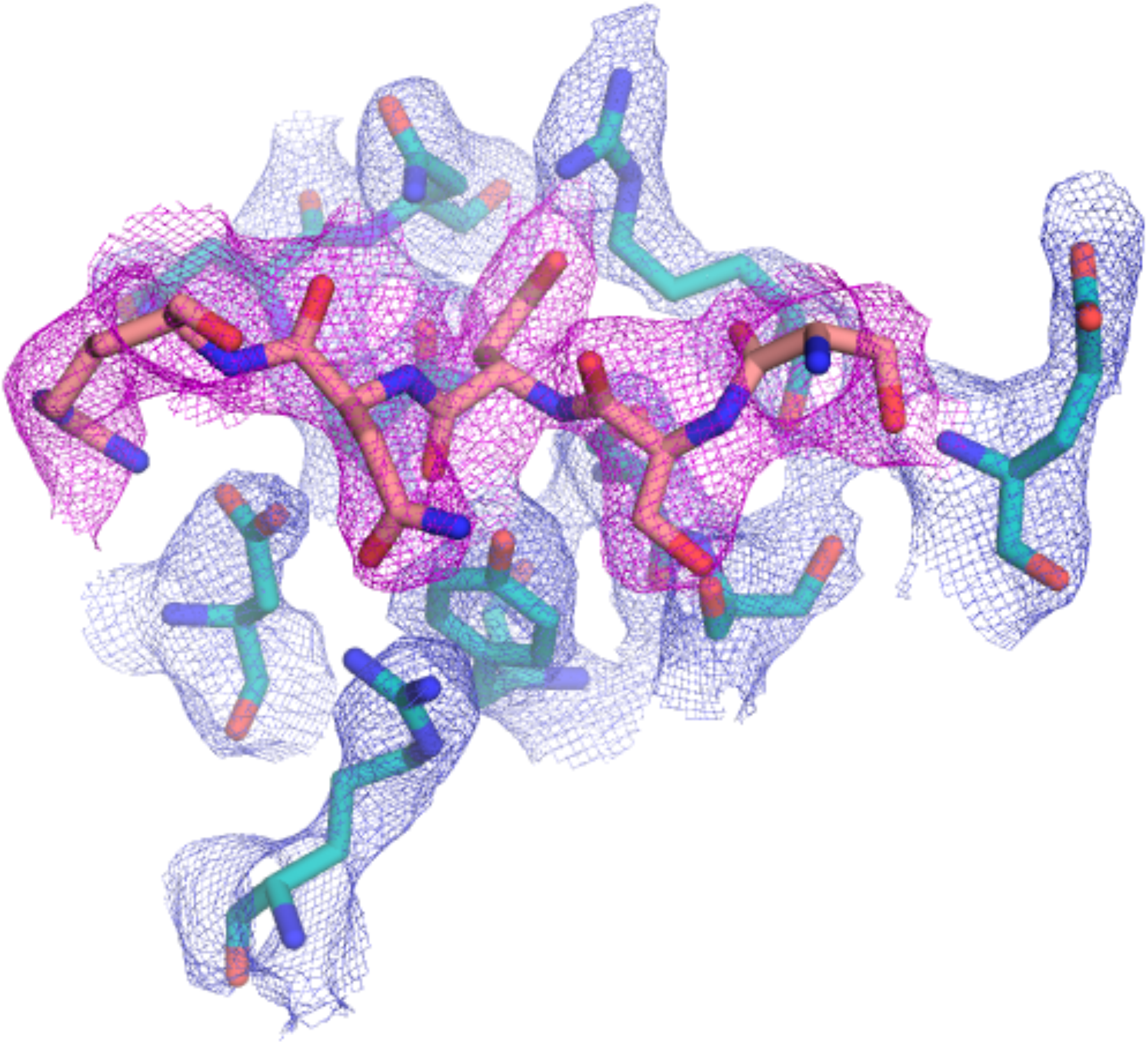

Supplementary Fig. 2. Electron density at the NKR-P1B:Clr-b interface. The $2F_O$-F_c electron density map (mesh) is shown contoured at 1σ and colored blue for Clr-b and pink for NKR-P1B. The orientation shown is the same as in Fig 3E.

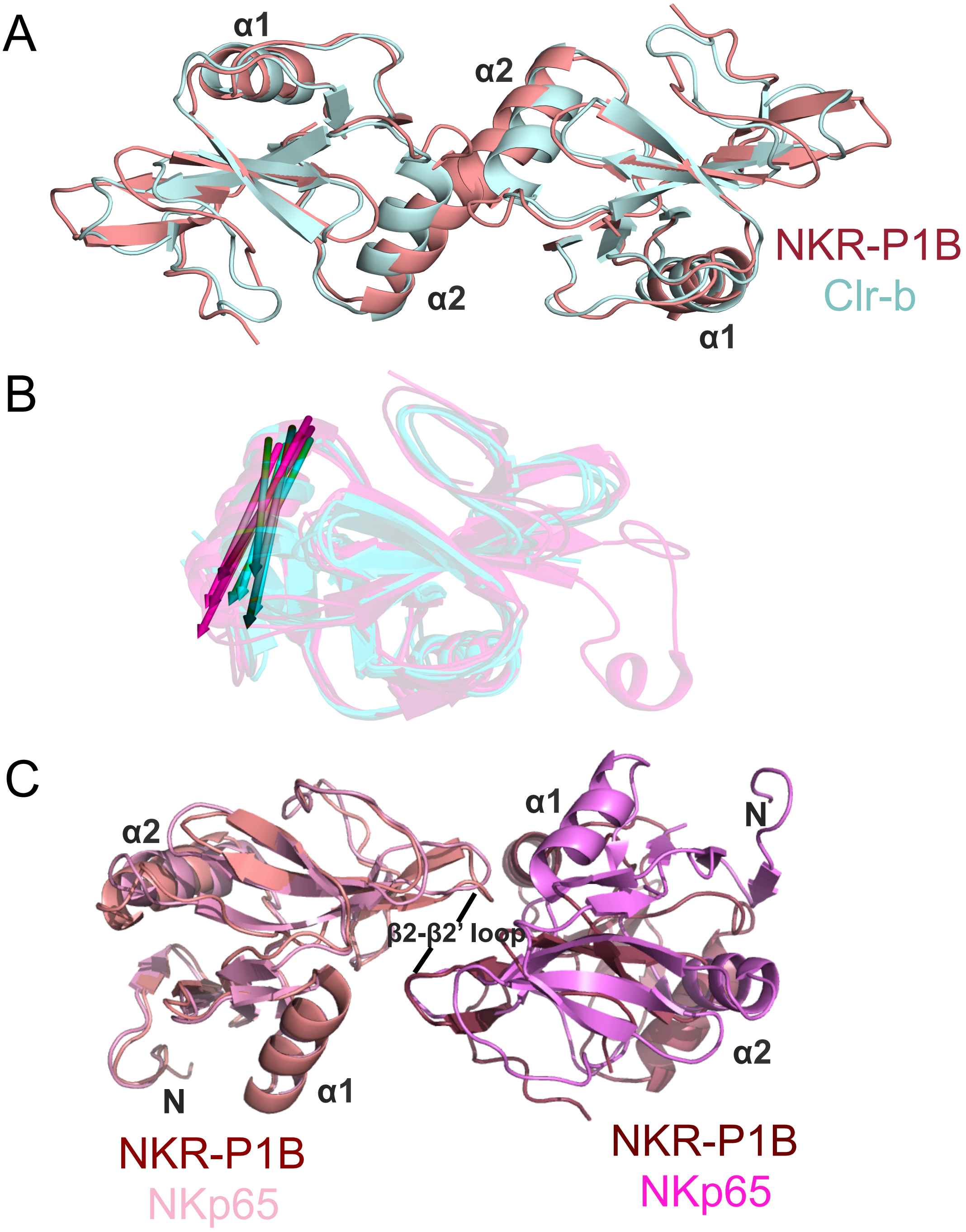

Supplementary Figure 3. **Homodimerization of NKR-P1B** (A) Overlay of two NKR-P1B monomers onto the Clr-b homodimer. The main structural difference between NKR-P1B and Clr-b - protrusion of the α2 helix in NKR-P1B- is incompatible with the classic CTLD homodimer. (B) Overlay of a variety of CTLD-containing receptors and ligands. Molecules that have been reported to form a 'classic' CTLD homodimer (LLT1, KACL, Clr-g and CD69) are shown in shades of cyan, while those that do not form such an assembly (NKR-P1A, CLEC9A and NKp65) are shown in shades of magenta. The center of gravity of the α2 helix is indicated by an arrow. (C) Comparison of the non-classical NKR-P1B homodimer to that of NKp65 (PDB ID: 4IOP). Each homodimer was aligned based only on the left-hand side protomer.

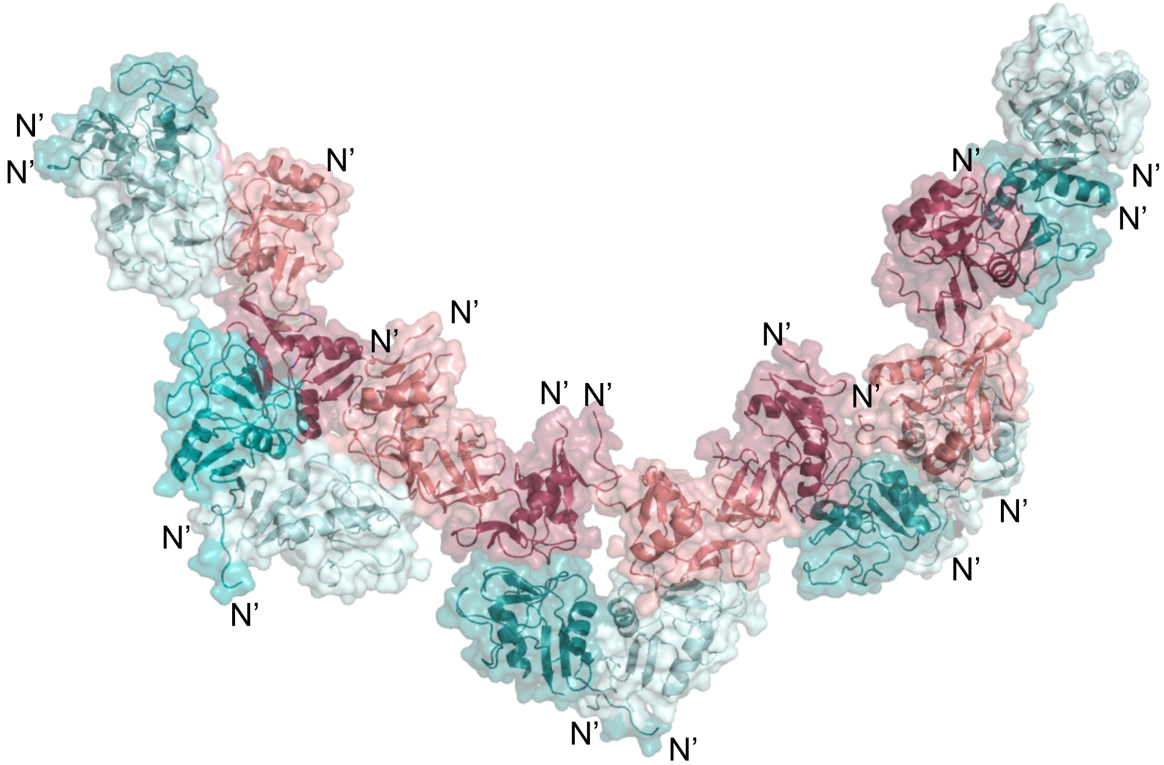

Supplementary Figure 4. Model of a fully saturated NKR-P1B:Clr-b assembly. The model was constructed using five classical Clr-b homodimers and fours non-classical NKR-P1B homodimers in a manner such that all the NKR-P1B:Clr-b binding sites were occupied. NKR-P1B and Clr-b are shown in shades of salmon and cyan respectively.

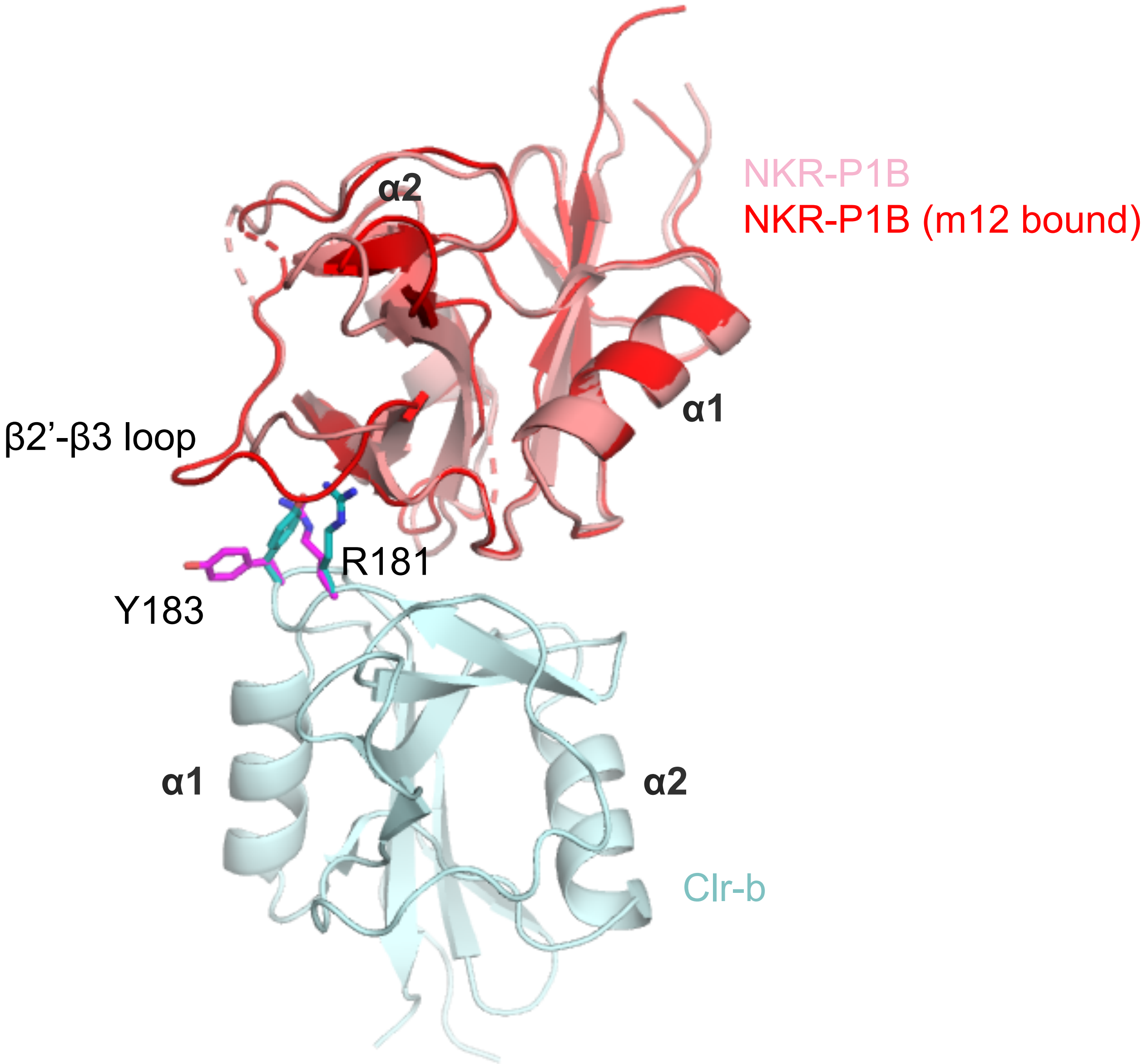

Supplementary Figure 5. **Plasticity at the NKR-P1B:Clr-b interface**. Overview of a monomer of NKR-P1B bound to a monomer of Clr-b. Side chain residues that change conformation relative to the unbound Clr-b structure (magenta) are shown as sticks. The structure of the m12-bound form of NKR-P1B (PDB ID: 5TZN) is also shown to highlight how the β2'-β3 loop remodels to accommodate Arg181 and Tyr183 of Clr-b.

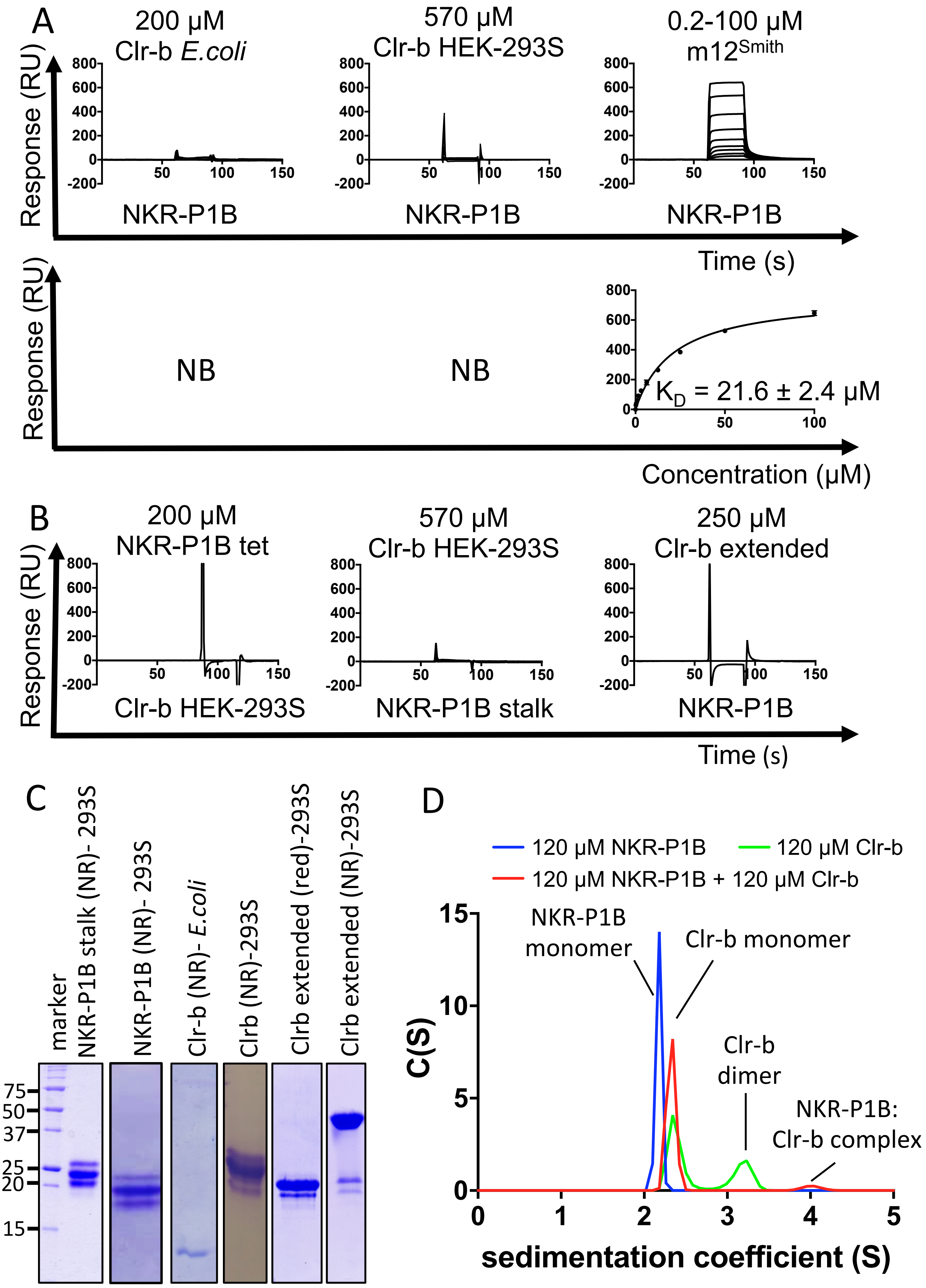

Supplementary Figure 6. Binding studies of NKR-P1B:Clr-b. (A) SPR analysis of

the NKR-P1B:Clrb interaction. Sensograms (top) and equilibrium binding curves (bottom) for the binding of m12^{Smith} and Clr-b to immobilized NKR-P1B are shown. Equilibrium binding curves represent the mean of 2 independent experiments, each performed in duplicate. Equilibrium dissociation constants (K_D) and standard error of the mean (depicted as error bars) were calculated from the independent experiments. NB indicates no binding. (B) SPR sensograms showing the response to a variety of different NKR-P1B and Clr-b constructs. For (A) and (B), the analyte and ligand are labeled above and below each corresponding sensogram, respectively. All SPR sensograms are representative of two independent experiments performed. (C) SDS-PAGE analysis of the various NKR-P1B and Clr-b constructs tested in SPR/AUC. Samples were visualized under non-reducing conditions (NR) or in the presence of 5mM β-mercaptoethanol (red) as indicated. (D) Sedimentation velocity analytical ultracentrifugation analysis of the binding of mammalian expressed NKR-P1B-stalk and Clr-b. The identity of each species in the C(S) profile is indicated.

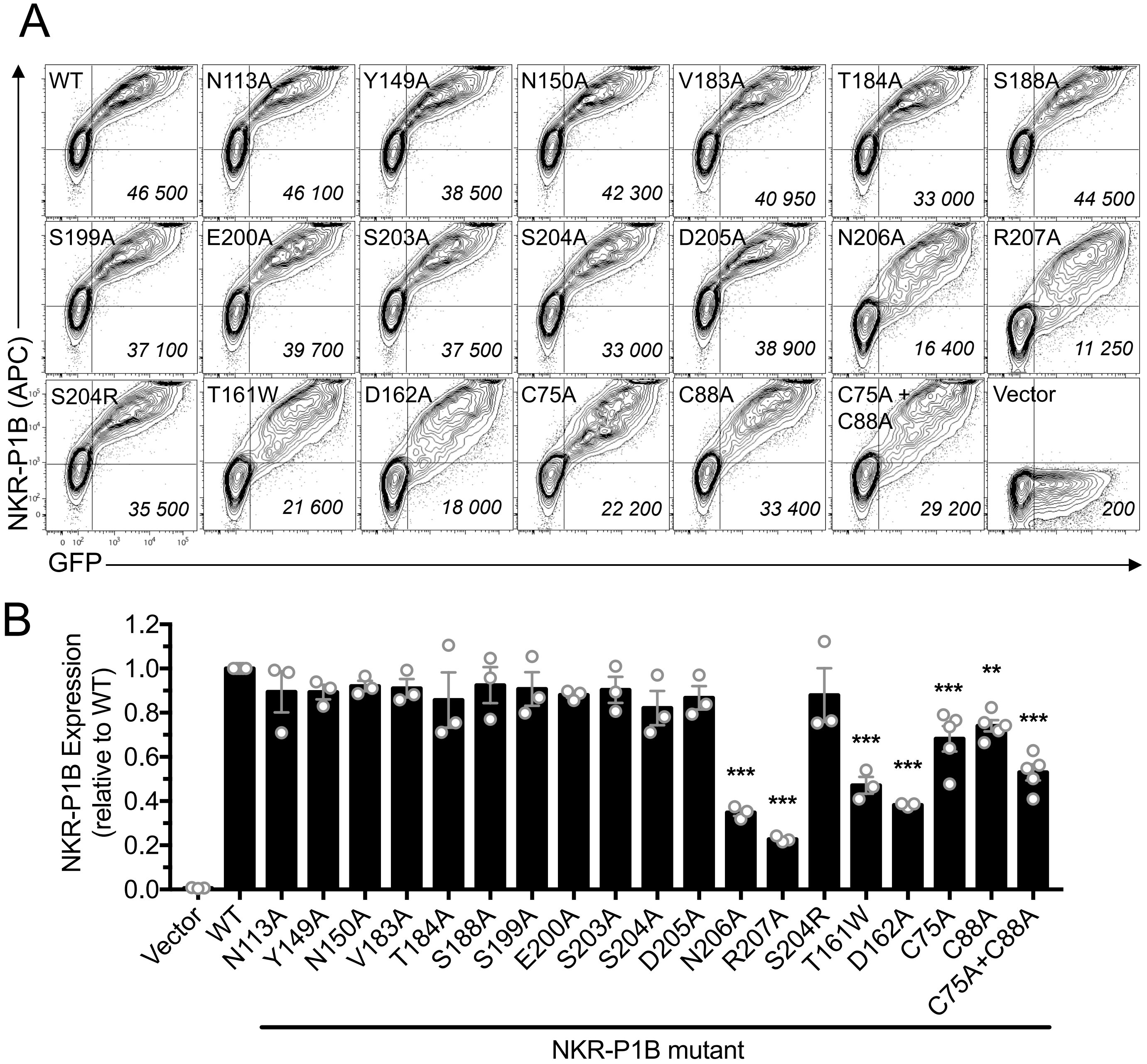

Supplementary Figure 7. Cell surface expression of the NKR-P1B mutants. (A) HEK293T cells were transfected with mammalian expression vector (pIRES2-EGFP) expressing the NKR-P1B mutants, and 48 hours later were analyzed by flow cytometry using the anti-NKR-P1B^{B6} mAb, 2D12. GFP is expressed by the vector and is used to demonstrate transfection efficiency. Gates were drawn based on untransfected cells (GFP^{-}/APC^{-}). The mutant residues are displayed on the top left of the plots, whereas the italicized numbers on the bottom right represent median fluorescence intensity values. (B) Graphical representation of relative NKR-P1B expression on transfected cells (GFP^{+}) in (A). Data were normalized to WT NKR-P1B expression and significant differences are shown between the WT and mutant allele for each graph. Data were analyzed using one-way ANOVA with Bonferroni post-hoc tests [$F(20,50) = 23.25$, $p < 0.0001$]. Significance intervals are depicted as ** $p < 0.01$, *** $p < 0.001$. All data are representative of at least 3 independent biological replicates. Data are presented as mean ± SEM.

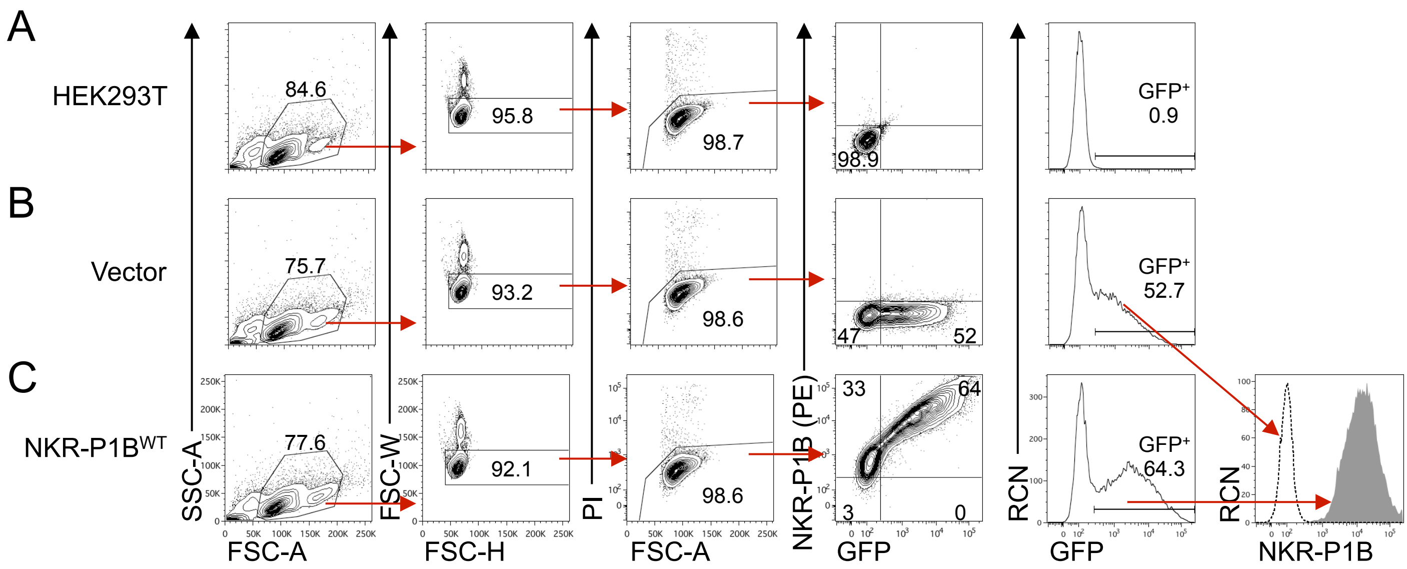

Supplementary Figure 8. Gating strategies for the flow cytometric analysis in this study. (A) Untransfected HEK293T cells and cells transfected with (B) empty pIRES2-EGFP vector or (C) NKR-P1B expressing vector were analyzed on flow cytometer. Untransfected HEK293T cells (top) were used to setup gates: cells were gated for doublet exclusion and viability (PI^-, propidium iodide negative), then GFP^+ and NKR-$P1B^+$ (biotinylated 2D12 + streptavidin PE) gates were determined. 2D plots are visualized according to GFP and NKR-P1B expression. For histogram overlays, the GFP+ gated cells from NKR-P1B transfectants were overlaid with vector controls (shown in D). Quantitation was obtained from GFP+ cells in all experiments.

Supplementary Tables

Supplementary Table 1. Contacts between Clr-b dimers.

Clr-b (Chain G)	Clr-b (Chain H)	Contact Type
Tyr77	Ala78, Ala79, Cys80,	VDW
Ala78	Tyr77, Ala78, Ala79,	VDW
Ala78	Ala79	H-Bond (x2)
Ala79	Glu88	VDW
Pro81	Glu88	VDW
Gln82	Glu88	H-Bond
Gln82	Glu88	VDW
Ile85	Ile85, Gly86	VDW
Gly86	Gly86	H-Bond (x2)
Gly86	Ile85, Gly86	VDW
Glu88	Ala78, Asn83	VDW
Glu88	Ala78	H-Bond
Asn89	Thr76, Tyr77	VDW
Phe94	Arg129	VDW
Phe126	Tyr130	VDW
Arg129	Tyr130	VDW
Arg129	Tyr130	H-Bond
Tyr130	Phe126, Arg129, Tyr130	VDW
Tyr130	Arg129	H-Bond
Lys131	Arg129	VDW
Lys131	Arg129	H-Bond
Ala132	Arg129	VDW
Phe134	Arg129	VDW
Asn194	Thr76, Tyr77, Ala79	VDW
Asn194	Tyr77	H-Bond
Ala195	Met73, Lys75, Tyr77	VDW

Atomic contacts were determined with CONTACT available within the CCP4i package. Van der Waals interactions were defined as non-hydrogen bonded contacts up to a distance of 4 Å. Hydrogen bond and salt bridge interactions were defined as contact distances of less than 3.5 Å or 4.5 Å respectively.

Supplementary Table 2. Contacts between NKR-P1B dimers.

NKR-P1B (Chain T)	NKR-P1B (Chain U)	Contact Type
Trp115	Thr161	VDW
Arg119	Asp162	Salt-Bridge
Ser157	Thr161	VDW
Thr159	Thr159	VDW
Thr161	Trp115, Tyr158, Thr159, Ser188, Cys189, Cys202	VDW
Asp162	Tyr115, Lys116	VDW
Asp162	Arg119	Salt-Bridge
Lys166	Arg119	VDW

Atomic contacts were determined with CONTACT available within the CCP4i package. Van der Waals interactions were defined as non-hydrogen bonded contacts up to a distance of 4 Å. Hydrogen bond and salt bridge interactions were defined as contact distances of less than 3.5 Å or 4.5 Å respectively.

Supplementary Table 3. Contacts between NKR-P1B and Clr-b.

**NKR-P1B**	**Clr-b**	**Contact Type**
Ser112	Asn175	VDW
Ser112	Asn175	H-bond
Asn113	Arg164	VDW
Tyr149	Tyr97, Pro98	VDW
Val183	Tyr183	VDW
Thr184	Arg181	H-bond
Thr184	Arg181, Tyr183	VDW
Asn186	Arg181	VDW
Ser188	Arg181	VDW
Ser188	Arg181	H-bond (x2)
Ser199	Arg181	H-bond
Ser199	Arg181, Tyr183	VDW
Ser199	Tyr183	H-bond
Glu200	Thr180, Arg181	VDW
Glu200	Ser184	VDW
Glu200	Ser184	H-bond
Glu200	Arg186	Salt-Bridge (x2)
Gly201	Arg181	H-bond (x2)
Gly201	Arg181	VDW
Ser203	Gly165, Glu166	VDW
Ser203	Glu166	H-bond
Ser204	Ser179, Thr180	VDW
Ser204	Ser179	H-bond
Asp205	Arg164, Tyr171, Asn173, Asn175, Ser178	VDW
Asp205	Arg164	Salt-Bridge (x2)
Asp205	Tyr171	H-bond (x2)
Asp205	Asn173	H-bond
Asp205	Asn175	H-bond
Asp205	Ser178	H-bond (x2)
Asn206	Arg186	VDW
Asn206	Arg186	H-bond
Arg207	Asp135, Asn174	VDW
Arg207	Asp135	Salt-Bridge (x4)

Atomic contacts were determined with CONTACT available within the CCP4i package. Van der Waals interactions were defined as non-hydrogen bonded contacts up to a distance of 4 Å. Hydrogen bond and salt bridge interactions were defined as contact distances of less than 3.5 Å or 4.5 Å respectively.

Supplementary Table 4. List of PCR primers used in this study.

Construction of Nkrp1b^{B6} mutants for reporter assays		
**Construct**	**Primer Name**	**Sequence (5'→3')**
NKR-P1B CDS	Nkrp1b_B6 FXATG	gaattcctcgagATGGATTCAACAACACTGGTC
	Nkrp1b_B6 RPTGA	aaaactgcagTCAGGAGTCATTACACGGG
NKR-P1B NT-FLAG tagged	Nkrp1b_B6 FLAG-FXATG	gaattcctcgagATGGACTACAAGGACGACGACGACAAGGGATCAGGATCAATGGATTCAACAACACTGGTC
Nkrp1b_N113A	1_N113A Fwd	CAAGTTTCCGCCACTTGGAAG
	1_N113A Rev	CTTCCAAGTGGCGGAAACTTG
Nkrp1b_Y149A	2_Y149A Fwd	GGAAAAAGCCAATTCATTTTGG
	2_Y149A Rev	CCAAAATGAATTGGCTTTTTCC
Nkrp1b_N150A	3_N150A Fwd	GGAAAAATACGCCTCATTTTGG
	3_N150A Rev	CCAAAATGAGGCGTATTTTTCC
Nkrp1b_V183A	4_V183A Fwd	CACTGGTGCCACTGAAAATGG
	4_V183A Rev	CCATTTTCAGTGGCACCAGTG
Nkrp1b_T184A	5_T184A Fwd	CACTGGTGTCGCCGAAAATGG
	5_T184A Rev	CCATTTTCGGCGACACCAGTG
Nkrp1b_S188A	6_S188A Rev1	GATCCAACGGTTGTCTGAAGAACAGCCCTCAGAAGTCACTTTTTCTCCTGAGATGGCGGCACAGGCGCC
	6_S188A Rev2	aaaactgcagTCAGGAGTCATTACACGGGGTTTCATGGTTTAGTTCCTTTTGGCAGATCCAACGGTTGTC
Nkrp1b_S199A	7_S199A Rev1	GGTTTAGTTCCTTTTGGCAGATCCAACGGTTGTCTGAAGAACAGCCCTCGGCAGTCAC
Nkrp1b_E200A	8_E200A Rev1	GGTTTAGTTCCTTTTGGCAGATCCAACGGTTGTCTGAAGAACAGCCGGCAGAAG
Nkrp1b_S203A	9_S203A Rev1	GGTTTAGTTCCTTTTGGCAGATCCAACGGTTGTCTGAGGCACAGCC
Nkrp1b_S204A	10_S204A Rev1	GGTTTAGTTCCTTTTGGCAGATCCAACGGTTGTCGGCAGAACAGCC
	7-10+15_S199-204 R2	aaaactgcagTCAGGAGTCATTACACGGGGTTTCATGGTTTAGTTCCTTTTGG
Nkrp1b_D205A	11_D205A Rev	aaaactgcagTCAGGAGTCATTACACGGGGTTTCATGGTTTAGTTCCTTTTGGCAGATCCAACGGTTGGCTGAAG
Nkrp1b_N206A	12_N206A Rev	aaaactgcagTCAGGAGTCATTACACGGGGTTTCATGGTTTAGTTCCTTTTGGCAGATCCAACGGCGTCTG
Nkrp1b_R207A	13_R207A Rev	aaaactgcagTCAGGAGTCATTACACGGGGTTTCATGGTTTAGTTCCTTTTGGCAGATCCAGGCGTTGTC
Nkrp1b_D162A	14_D162A Fwd	CATTAACAGCCATGAACTGG
	14_D162A Rev	CCAGTTCATGGCTGTTAATG
Nkrp1b_S204R	15_S204R Rev1	CATGGTTTAGTTCCTTTTGGCAGATCCAACGGTTGTCCCTAGAACAGCC
Nkrp1b_T161W	23_T161W Fwd	ACACATTAtggGACATGAACTG
	23_T161W Rev	CAGTTCATGTCccaTAATGTGT
Nkrp1b_C75A	24_C75A Fwd	GTACAAAAAATCGCCGCGGATGTTC
	24_C75A Rev	GAACATCCGCGGCGATTTTTTGTAC
Nkrp1b_C88A	25_C88A Fwd	CATACAACAGGTGCCTCAGCTAAGC
	25_C88A Rev	GCTTAGCTGAGGCACCTGTTGTATG

Nkrp1b_C75+88A	26_C75/88A Fwd	TCGCCGCGGATGTTCAAGAGAACAGAACACATACAACAG GTGCCTC
	26_C75/88A Rev	GAGGCACCTGTTGTATGTGTTCTGTTCTCTTGAACATCCG CGGCGA
Cloning of Clr-b		
**Construct**	**Primer Name**	**Sequence (5'→3')**
Clr-b CDS	Clrb FXATG	gaattcctcgagATGTGTGTCACAAAGGCTTCC
	Clrb RBTGA	gcgaaggatccCTAGGAAGGAAAAAAAGGAGTT
Generation of CD3ζ-Clr-b Type II MSCV vector		
**Construct**	**Primer Name**	**Sequence (5'→3')**
Clr-b ectodomain	Clr-b EC X-Fwd	gaattcctcgagAAGACAGAACAGATCCCAG
	Clr-b EC N-Rev	ataagaatgcggccgcCTAGGAAGGAAAAAAAGGAG
Construction of Nkrp1b^{B6} mutants for tetramer staining		
**Construct**	**Primer Name**	**Sequence (5'→3')**
NKR-P1B^{B6} N113A	N113A forward	GTGAGCCAGGTATCCGCTACTTGGAAAGAGGG
NKR-P1B^{B6} N113A	N113A reverse	CCCTCTTTCCAAGTAGCGGATACCTGGCTCAC
NKR-P1B^{B6} N149A	N149A forward	GCATTAAAGAGAAAGCTAATTCATTTTGGATTG
NKR-P1B^{B6} N149A	N149A reverse	CAATCCAAAATGAATTAGCTTTCTCTTTAATGC
NKR-P1B^{B6} N150A	N150A forward	CATTAAAGAGAAATATGCTTCATTTTGGATTGG
NKR-P1B^{B6} N150A	N150A reverse	CCAATCCAAAATGAAGCATATTTCTCTTTAATG
NKR-P1B^{B6} D162A	D162A forward	CATATACATTGACCGCCATGAACTGGAAGTGG
NKR-P1B^{B6} D162A	D162A reverse	CCACTTCCAGTTCATGGCGGTCAATGTATATG
NKR-P1B^{B6} V183A	V183A forward	GAAGATCACAGGTGCCACTGAAAATGGCAG
NKR-P1B^{B6} V183A	V183A reverse	GCCATTTTCAGTGGCACCTGTGATCTTCAG
NKR-P1B^{B6} T184A	T184A forward	GAAGATCACAGGTGTCGCTGAAAATGGCAGTTGTG
NKR-P1B^{B6} T184A	T184A reverse	CAACTGCCATTTTCAGCGACACCTGTGATCTTC
NKR-P1B^{B6} S188A	S188A forward	GTCACTGAAAATGGCGCTTGTGCTGCCATAAG
NKR-P1B^{B6} S188A	S188A reverse	CTTATGGCAGCACAAGCGCCATTTTCAGTGAC
NKR-P1B^{B6} S199A	S199A forward	GGGGAGAAGGTCACCGCAGAAGGCTGCTCATC
NKR-P1B^{B6} S199A	S199A reverse	GATGAGCAGCCTTCTGCGGTGACCTTCTCCCC
NKR-P1B^{B6} E200A	E200A forward	GAGAAGGTCACCTCAGCAGGCTGCTCATCAGATAAC
NKR-P1B^{B6} E200A	E200A reverse	GTTATCTGATGAGCAGCCTGCTGAGGTGACCTTCTC
NKR-P1B^{B6} S203A	S203A forward	CCTCAGAAGGCTGCGCATCAGATAACAGGTGG
NKR-P1B^{B6} S203A	S203A reverse	CCACCTGTTATCTGATGCGCAGCCTTCTGAGG
NKR-P1B^{B6} S204A	S204A forward	CTCAGAAGGCTGCTCAGCAGATAACAGGTGGATATG
NKR-P1B^{B6} S204A	S204A reverse	CATATCCACCTGTTATCTGCTGAGCAGCCTTCTGAG
NKR-P1B^{B6} D205A	D205A forward	GAAGGCTGCTCATCAGCTAACAGGTGGATATG
NKR-P1B^{B6} D205A	D205A reverse	GCATATCCACCTGTTAGCTGATGAGCAGCCTTC
NKR-P1B^{B6} N206A	N206A forward	GGCTGCTCATCAGATGCTAGGTGGATATGCCAG
NKR-P1B^{B6} N206A	N206A reverse	CTGGCATATCCACCTAGCATCTGATGAGCAGCC
NKR-P1B^{B6} R207A	R207A forward	GCTCATCAGATAACGCGTGGATATGCCAGAAG
NKR-P1B^{B6} R207A	R207A reverse	CTTCTGGCATATCCACGCGTTATCTGATGAGC
NKR-P1B^{B6} S204R	S204R forward	CAGAAGGCTGCTCAAGAGATAACAGGTGGATATG
NKR-P1B^{B6} S204R	S204R reverse	CATATCCACCTGTTATCTCTTGAGCAGCCTTCTG
Generation of extended NKR-P1B		
**Construct**	**Primer Name**	**Sequence (5'→3')**
NKR-P1B extended	NKR-P1B ext	CTCGTCGGTACCACTATCATTGCAAGG<br>GGTCTCATGGTTCAGCTCCTTCTGGCATATC

Generation of extended Clr-b		
**Construct**	**Primer Name**	**Sequence (5'→3')**
Clr-b extended	Clr-b S207 Rev	CTCGTCCTCGAGTTAGGTACCGCTCGGA AAGAAAGGAGTCTGGCAATGAAGAGAG TAAGAGTTCAGTTTTGAGCAAATCCAC
